# Update of the trauma risk adjustment model of the TraumaRegister DGU™: the Revised Injury Severity Classification, version II

**DOI:** 10.1186/s13054-014-0476-2

**Published:** 2014-09-05

**Authors:** Rolf Lefering, Stefan Huber-Wagner, Ulrike Nienaber, Marc Maegele, Bertil Bouillon

**Affiliations:** Institute for Research in Operative Medicine (IFOM), University of Witten/Herdecke, Ostmerheimer Strasse 200 (Building 38), 51109 Cologne, Germany; Department of Trauma Surgery, Technical University Munich, Hospital Rechts der Isar, Ismaninger Strasse 22, 81675 Munich, Germany; AUC - Academy for Trauma Surgery, Ostmerheimer Strasse 200, 51109 Cologne, Germany; Department of Trauma and Orthopaedic Surgery, University of Witten/Herdecke, Cologne-Merheim Medical Center, Ostmerheimer Strasse 200, 51109 Cologne, Germany

## Abstract

**Introduction:**

The TraumaRegister DGU™ (TR-DGU) has used the Revised Injury Severity Classification (RISC) score for outcome adjustment since 2003. In recent years, however, the observed mortality rate has fallen to about 2% below the prognosis, and it was felt that further prognostic factors, like pupil size and reaction, should be included as well. Finally, an increasing number of cases did not receive a RISC prognosis due to the missing values. Therefore, there was a need for an updated model for risk of death prediction in severely injured patients to be developed and validated using the most recent data.

**Methods:**

The TR-DGU has been collecting data from severely injured patients since 1993. All injuries are coded according to the Abbreviated Injury Scale (AIS, version 2008). Severely injured patients from Europe (ISS ≥4) documented between 2010 and 2011 were selected for developing the new score (n = 30,866), and 21,918 patients from 2012 were used for validation. Age and injury codes were required, and transferred patients were excluded. Logistic regression analysis was applied with hospital mortality as the dependent variable. Results were evaluated in terms of discrimination (area under the receiver operating characteristic curve, AUC), precision (observed versus predicted mortality), and calibration (Hosmer-Lemeshow goodness-of-fit statistic).

**Results:**

The mean age of the development population was 47.3 years; 71.6% were males, and the average ISS was 19.3 points. Hospital mortality rate was 11.5% in this group. The new RISC II model consists of the following predictors: worst and second-worst injury (AIS severity level), head injury, age, sex, pupil reactivity and size, pre-injury health status, blood pressure, acidosis (base deficit), coagulation, haemoglobin, and cardiopulmonary resuscitation. Missing values are included as a separate category for every variable. In the development and the validation dataset, the new RISC II outperformed the original RISC score, for example AUC in the development dataset 0.953 versus 0.939.

**Conclusions:**

The updated RISC II prognostic score has several advantages over the previous RISC model. Discrimination, precision and calibration are improved, and patients with partial missing values could now be included. Results were confirmed in a validation dataset.

## Introduction

Severe trauma has serious consequences for the victims with a still considerable mortality rate and often long-lasting physical and mental problems for the survivors. But it is also a serious public health problem since most trauma victims are young and the accident impairs their role in society, work, and families in multiple ways. Therefore, improvement of quality of care and reduction of mortality and morbidity in severe trauma cases is an important aim of health care policy. Trauma registries are able to provide an important contribution to quality assessment and scientific research in the area of acute care where classical randomized trials are difficult to perform. Benchmarking of hospital results needs to consider the case mix and the injury pattern, and scientific analyses have to deal with the comparability of study groups. In order to reach these aims, it is absolutely necessary to be able to accurately describe injury severity, or the risk of death, on an individual basis. Only a valid estimation of baseline risk allows interpretation of observed mortality rates. This is underlined by a statement from Susan Baker, who published the Injury Severity Score (ISS): *‘*If you have never felt the need for any type of severity scoring system, then you probably have never had to explain how it is that survival rate of 85% in your trauma center is actually better than the survival rate of 97% in some other hospital where the patients are much less seriously injured*’* [1].

In the history of trauma severity scores the description of anatomical injuries was the starting point. The publication of the ISS was a landmark article, and still today this score is the most frequently used trauma score worldwide [2]. But already in the 1980s, it became clear that the patient’s physiological response to an injury, as well as age, are important predictors of outcome, too. The Trauma and Injury Severity Score (TRISS) developed with data from the Major Trauma Outcome Study did consider these aspects, and it became the most frequently used tool for outcome adjustment and benchmarking in trauma registries [3].

The TraumaRegister DGU™ of the German Trauma Society (Deutsche Gesellschaft für Unfallchirurgie (DGU)) (TR-DGU), a national initiative for documentation of care of severely injured patients in Germany, founded in 1993, also used the TRISS for inter-hospital comparisons. However, in 2003 a new risk adjustment model developed with data from the registry was introduced, the Revised Injury Severity Classification (RISC) score [4]. For the first time, initial laboratory values were included in the score (base deficit, hemoglobin, partial thromboplastin time) as well as interventions (cardiopulmonary resuscitation (CPR)). This allowed describing the patients’ condition and prognosis on admission more precisely. Discrimination, precision, and calibration improved, compared to the previously used TRISS model, even if the TRISS coefficients were adapted to the registry data. In the following years, the RISC has repeatedly been validated with TR-DGU data.

However, during recent years some limitations of the RISC have also become apparent. It uses 10 different variables for prediction, which makes it increasingly difficult to provide complete data in all patients. Complete data were available for only about 25% of cases. The existing procedure to replace missing values is complex and difficult to use. But despite these efforts, the percentage of patients with an available RISC prognosis repeatedly fell below the desired rate of 90%. Thus a considerable amount of patients could not be included in comparative analyses. Furthermore, the RISC had been developed with data from 1993 to 2000, which led to an overestimation of risk of death in recent years. Since 2006 the observed mortality was about 2% below the predicted one.

These reasons led us to revise and update the RISC score, with the aim to establish a more accurate, up-to-date, and easier to use model for risk of death estimation in severely injured patients.

## Materials and methods

### TraumaRegister DGU™

The TR-DGU was founded in 1993. The aim of this multicentre database was an anonymous and standardized documentation of severely injured patients for benchmarking of hospitals and health services research in the field of severe trauma.

Data are collected from four consecutive time phases from the site of the accident until discharge from hospital: (A) pre-hospital phase, (B) emergency room and initial surgery, (C) intensive care unit and (D) discharge and outcome. The documentation includes detailed information on demographics, injury pattern, comorbidities, pre- and in-hospital management, time course, relevant laboratory findings including data on transfusion and outcome of each individual. The inclusion criterion is admission to hospital with vital signs via the emergency room with subsequent intensive care treatment, including those who die before admission to the intensive care unit.

The infrastructure for documentation, data management and data analysis is provided by the Academy for Trauma Surgery (Akademie der Unfallchirurgie GmbH (AUC)), a company of the DGU. The scientific leadership is provided by the Committee on Emergency Medicine, Intensive Care and Trauma Management (Sektion NIS) of the German Trauma Society (DGU). The participating hospitals submit their data anonymously into a central database via a web-based application. Multiple plausibility checks have been implemented into this application in order to improve data quality.

Participation in the TR-DGU is voluntary, but for certified trauma centres associated with the TraumaNetzwerk DGU™ (the German Trauma Network, an initiative of the DGU to establish local networks of hospitals involved in trauma care) participation is obligatory. Hospitals certified as a member of a regional trauma network but not interested in trauma research could chose a reduced data collection form with only 40 items per case while the standard data collection form contains about 100 items. Both data forms are in compliance with the European Core Dataset (Utstein Template, see [5]). All hospitals receive extended annual audit reports. As a compulsory tool for quality assessment in certified regional trauma networks, which is based on routinely available data only, no informed consent is necessary for data collection. However, no personal data are collected in the registry, and only the hospital is able to re-identify a certain case for the purpose of internal audits. Completeness of cases is a prerequisite for a meaningful evaluation of a hospital’s quality. The hospitals have permission for participation from their local authorities. All scientific analyses using registry data are based on anonymous data. The process is managed by the Sektion NIS in cooperation with the AUC; it is based on a publication guideline approved by the scientific society DGU. This guideline has to be signed for and followed by each researcher. The present analysis has been registered, evaluated, and approved by the internal review board of the Sektion NIS (No. 2013–006). Detailed information including the data collection forms in German and English, the participating hospitals, and the publication guideline are available at the registry’s homepage [6].

In 2012, a total of 28,805 severely injured patients from 572 different hospitals were documented in the TR-DGU. The patients primarily came from Germany (90%), but other countries contribute data as well (Austria, Belgium, China, Finland, Luxembourg, Slovenia, Switzerland, The Netherlands, and the United Arab Emirates).

### Patients

**Figure 1:**
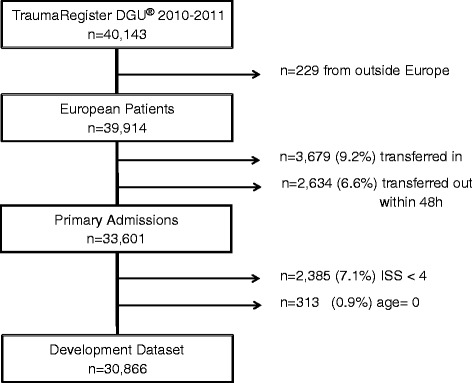
**Flow sheet for patient inclusion.**

Only European patients documented in 2010 or 2011 qualified for analysis (n = 39,914). Patients transferred to the reporting hospital after initial treatment in another hospital (n = 3,679; 9.2%) had to be excluded since the initial status on admission was unknown. Furthermore, primary admitted patients who had been transferred out into another hospital within 48 hours (n = 2,634; 6.6%) were excluded as well since their final outcome was considered unknown. Although the primary focus of the TR-DGU is on severely injured patients, only about half of all documented patients fulfilled the ISS ≥16 criterion. Since the RISC score should cover the large majority of the registry patients, it was decided to only exclude cases with a worst injury of AIS grade 1 (n = 2,385). In these patients, risk of death estimation is considered inappropriate since virtually all of them survive. This means that the ISS has to be at least 4 points. Finally, there has to be a valid entry for age. Since age is a compulsory variable every patient has a documented age, which is calculated from the date of birth (which is entered but not stored) and the date of accident. However, mistyping the actual year instead of the year of birth results in an age of zero, which is six times more frequent in the database than the age of one year. Therefore, patients with an age of zero were excluded (n = 313). This leaves a total of 30,866 cases (77%) for final analysis (Figure 1).

The internal validation of the new model was performed on TR-DGU patients documented in 2012, using the same inclusion and exclusion criteria as described above, that is European patients with ISS ≥4 points and age >0, except transfers (n = 21,918 of 28,805, 76%).

### Trauma scores

All injuries are coded according to the Abbreviated Injury Scale (AIS), version 2008. The AIS codebook contains about 2000 different injuries, each one with an individual severity level ranging from 1 (minor) to 6 (actual untreatable). The TR-DGU uses a reduced version with only 450 codes for documentation where similar codes with the same severity level were merged.

The ISS is calculated from the three worst affected body regions as the sum of squares of the respective AIS severity levels [2]. The New ISS, or NISS, is calculated in a similar way but here the three worst injuries are selected regardless of their location [7]. The TRISS is a combination of anatomical injury severity (ISS), the physiological response (Revised Trauma Score with consciousness, blood pressure, and respiratory rate), and age. The TRISS has different formulas for blunt and penetrating trauma mechanism. This score has repeatedly been used and adapted to local trauma registries but we used the original coefficients of Champion *et al*. in this analysis for reasons of comparability [3].

The RISC score has been developed with about 1,200 cases from the TR-DGU documented in the years 1993 to 2000. Besides the NISS the following categorical variables were used in the RISC: age, head injury, Glasgow Coma Scale (GCS), coagulation (partial thromboplastin time), base deficit, CPR, number of indirect signs of bleeding (low haemoglobin; hypotension, massive transfusion). For most variables, an algorithm for replacing missing values had been established (for details, see [4]).

### Statistical analysis

Binary logistic regression analysis was used to derive the new score. Survival until discharge, or hospital mortality, was used as dependent variable, and various combination of potential predictor variables were used to create the model. The final score X of the model (the logit, or the natural logarithm of the odds of the dependent variable occurring or not) could then be transformed into a probability of survival using the logistic function:

$$ P\ (survival)\kern0.5em =\kern0.5em \frac{1}{1\kern0.5em +\kern0.5em  exp\ \left( - X\ \right)} $$

The score value X = 0 corresponds to a 50% probability for survival, while positive and negative values describe a better or worse prognosis, respectively. Age and injury severity (AIS codes) were required to have no missing data (see inclusion criteria) but all other predictor variables had a varying degree of missing values ranging from <1% (sex) to 55% (pupil size). A basic principle of the present analysis was not to impute or replace missing values, nor to exclude cases with missing data. ‘Missing value’ was rather included as a separate category of each variable in the model. This category was assigned the reference category, so that the coefficient for this category was automatically set to zero. This means that a missing value would not change the prognosis. Since the stability of the calculated coefficients and the odds ratios (OR) depend on the size of the reference category, it was decided to have at least 20% of cases in this category. In variables with less than 20% missing data, a randomly selected number of cases was assigned this category (that is their observed value was deleted during model building) so that the reference category comprised of about 20% of cases. It was required that the 95% confidence intervals (CIs) for mortality in all reference groups with missing values cover the overall mortality rate, which was found to be true for all variables.

All variables were included as categorical variables. Categories were built prior to analysis based on clinical judgement. If during model building a category showed no or only minor effects, this category was merged with the reference category.

Model building started with a basic model that included only age and injury severity as independent predictors. Other candidate variables were then checked one by one for improvement of Nagelkerke’s R^2^, a measure of strength of association of the model with the observed outcome. The final model was then built using all candidate variables, which proved to have additional predictive power. Repeated modelling then gave the final list of variables with an optimal number of categories per variable.

The quality of the final model, as well as the quality of the existing trauma scores, was evaluated in terms of discrimination, precision, and calibration. *Discrimination* measures the ability of a score to separate survivors from non-survivors. This is best summarized by calculating sensitivity and specificity for all potential cutoff points of the score. These values are summarized in a receiver operating characteristic (ROC) curve. The area under the ROC curve (AUC) varies between 0.5 (discrimination by chance) and 1.0 (perfect separation of survivor and non-survivor). The AUC is presented with its 95% CI. *Precision* describes the agreement of observed mortality rate and score-based prognosis. Finally, *calibration* is evaluated with the goodness-of-fit statistic of the Hosmer-Lemeshow statistic (HL). For this statistic, the whole population is split in deciles of approximately equal size. Observed and expected number of deaths is determined in each subgroup and then combined to give a chi-squared distributed statistic. Low values of the HL statistic indicate a good calibration.

Descriptive statistics are provided as counts and percentages for categorical variables, and mean, median and standard deviation (SD) for continuous variables. Significance testing was largely avoided since in large samples like this one even minor differences become statistically significant. All analyses have been performed with SPSS statistical software, version 21 (IBM Corp, Armonk, NY, USA).

## Results

Descriptive data of patients selected for analysis are given in Table 1. The population is typical for a western European trauma population, with 5% penetrating trauma. Hospital mortality was 11.5% in the whole study group, and 19.1% in the subgroup of patients with ISS ≥16. Patients were treated in 510 different hospitals, 108 of them (21%) were classified as supra-regional level 1 trauma centres. These hospitals provided 59% of all patients (Table 1).

**Table 1:** **Descriptive data of the development and the validation dataset**

	**Development dataset**	**Validation dataset**
Year of accident	2010 - 2011	2012
Number of cases	30,866	21,918
Age	47.3/47 (21.7)	48.4/48 (21.9)
Male sex	21,899 (71.6%)	15,430 (70.4%)
Blunt mechanism	27,701 (95.5%)	19,720 (94.9%)
Head injury (AIS ≥3)	10,498 (34.0%)	6,791 (31.0%)
Chest injury (AIS ≥3)	12,126 (39.3%)	8,022 (36.6%)
Abdominal injury (AIS ≥3)	3,375 (10.9%)	2,136 (9.7)
Injury of extremities and pelvic ring (AIS ≥3)	8,252 (26.7%)	5,390 (24.6%)
Injury Severity Score (ISS)	19.3/17 (13.1)	18.0/16 (12.6)
New ISS	24.1/22 (15.8)	22.6/17 (15.4)
ISS ≥16	17,045 (55.2%)	10,995 (50.2%)
Type of injury		
- traffic: car	7,183 (23.3%)	4.999 (22.8%)
- traffic: motorbike	4,129 (13.4%)	2,859 (13.0%)
- traffic: bicycle	2,492 (8.1%)	1,907 (8.7%)
- traffic: pedestrian	2,066 (6.7%)	1,476 (6.7%)
- high fall (>3 m)	5,086 (16.5%)	3,389 (15.5%)
- low fall (<3 m)	5,339 (17.3%)	4,171 (19.0%)
Length of stay in hospital (days)	17.9/13 (18.8)	16.7/12 (17.5)
Hospital mortality	3,557 (11.5%)	2,378 (10.8%)
RISC score available	26,041 (84.4%)	19,501 (89.0%)
TRISS score available	17,411 (56.4%)	12,450 (56.8%)
Transportation to hospital by helicopter	7,297 (24.3%)	4,528 (21.2%)
Hospital level of care		
- supra-regional trauma centre (level 1)	18,119 (58.7%)	12,556 (57.3%)
- regional trauma centre (level 2)	10,439 (33.8%)	7,427 (33.9%)
- local trauma centre (level 3)	2,309 (7.5%)	1,935 (8.8%)

Continuous variables given as mean/median (standard deviation (SD)); categorical variables as number of cases and percentage. AIS, Abbreviated Injury Scale; ISS, Injury Severity Score; NISS, New Injury Severity Score; RISC, Revised Injury Severity Classification; TRISS, Trauma and Injury Severity Score.

Model building started with injury severity. Several potential representations of injury severity were compared using Nagelkerke’s R^2^. The ISS as a continuous variable reached R^2^ = 0.257 while the NISS reached 0.322. The worst AIS severity score (categories 2 to 6) as a single predictor reached the same level (R^2^ = 0.322). If the second-worst injury was added, R^2^ increased to 0.367, which could further be improved by additional consideration of head injury (R^2^ = 0.386). This is only marginally lower than the maximum value observed for 10 categorical variables each representing one body region (according to the first digit of the AIS code, R^2^ = 0.389).

Age groups were added to the model with intervals of 5 years. It turned out that beginning with the age of 55, there was a significant increase in mortality. Subgroups above the age of 85 were merged due to the limited sample size. It was also found that children (age 1 to 10) had a better chance of survival than adolescents and adults. This basic model with injury severity and age showed already a considerable association with outcome (R^2^ = 0.457).

Further model building included only categorical variables in which the reference category (missing value) comprised of at least 20% of cases. Table 2 describes the 13 variables included in the final RISC II model, and Table 3 presents the model. The score points are rounded to one decimal. The overall association with outcome was R^2^ = 0.595.

**Table 2:** **Variables included in the final model of RISC II, and prevalence of missing values**

**Variable**	**Description**	**Missing values**
**Worst injury, second-worst injury**	AIS injury severity level; if only one injury was coded, the second-worst injury was set to zero	0%
**Head injury**	AIS injury severity level of the body region ‘head’ as defined for the ISS score	0%
**Age**	Age in years at the time of accident, 10 categories	0%
**Sex**	Males/females	0.9%
**ASA**	Pre-trauma ASA (American Society of Anesthesiologists) score, as defined in the Utstein core dataset [5]	16.3%
**Pupil reactivity**	Three categories according to the Eppendorf-Cologne Scale (ECS) [12]: brisk, sluggish, and none. The first pre-hospital assessment was used; if missing; assessment on admission was used	53.5%*
**Pupil size**	Three categories according to the Eppendorf-Cologne Scale [12]: normal, anisocoria, and bilateral dilated. The first pre-hospital assessment was used; if missing; assessment on admission was used	54.9%*
**Motor function**	The motor function was derived from the Glasgow Coma Store (GCS) motor score according to the Eppendorf-Cologne scale [12]: normal (6 points in GCS); directed (4-5); non-directed (2-3), and none (1). The first pre-hospital assessment was used if available; if missing assessment on admission was used in non-intubated cases	2.7%
**Mechanism**	Blunt or penetrating mechanism of injury	6.1%
**Blood pressure**	Systolic blood pressure (mmHg), first measurement after admission; in case of missing values the first pre-hospital measurement was used	10.7%
**Coagulation: INR**	International normalized ratio (INR); first measurement after admission	12.9%
**Acidosis: base deficit**	Base deficit, or base excess (mEq/l); first measurement after admission	46.0%
**Blood: haemoglobin**	Haemoglobin (g/dl); first measurement after admission	7.6%
**CPR**	Cardiopulmonary resuscitation (CPR), performed pre-hospitally in case of cardiac arrest (not in the emergency room)	3.2%

*Actually not part of the reduced data collection form used in 47.4% of cases. RISC II, Revised Injury Severity Classification II.

**Table 3:** **The RISC II model for prediction of mortality after trauma**

**Variable**	**Category**	**Score**	**Coefficient**	***P*** **value**	**OR**	**CI of OR**
**Worst injury**	2*	**0**	0		1	
	3	**−0.5**	−0.517	<0.001	0.60	0.46-0.78
	4	**−1.3**	−1,259	<0.001	0.28	0.22-0.38
	5	**−1.7**	−1.742	<0.001	0.18	0.13-0.23
	6	**−2.9**	−2.941	<0.001	0.05	0.03-0.09
**Second-worst injury**	0-2	**+0.2**	0.254	<0.001	1.29	1.13-1.48
	3*	**0**	0		1	
	4	**−0.6**	−0.617	<0.001	0.54	0.47-0.62
	5-6	**−1.4**	−1.429	<0.001	0.24	0.20-0.29
**Head injury**	0-2*	**0**	0		1	
	3-4	**−0.2**	−0.199	0.004	0.82	0.72-0.94
	5-6	**−0.8**	−0.755	<0.001	0.47	0.40-0.56
**Age**	1-5	**+1.4**	1.411	0.001	4.10	1.79-9.40
	6-10	**+0.6**	0.581	0.099	1.79	0.90-3.57
	11-54*	**0**	0		1	
	55-59	**−0.5**	−0.544	<0.001	0.58	0.47-0.73
	60-64	**−0.8**	−0.784	<0.001	0.46	0.37-0.57
	65-69	**−0.9**	−0.921	<0.001	0.40	0.32-0.49
	70-74	**−1.2**	−1.250	<0.001	0.29	0.24-0.34
	75-79	**−1.9**	−1.888	<0.001	0.15	0.13-0.18
	80-84	**−2.4**	−2.244	<0.001	0.09	0.07-0.11
	85+	**−2.7**	−2.665	<0.001	0.07	0.06-0.08
**Sex**	???/males*	**0**	0		1	
	females	**+0.2**	0.231	<0.001	1.26	1.12-1.42
**ASA**	1-2	**+0.3**	0.335	<0.001	1.40	1.26-1.56
	???/3*	**0**	0		1	
	4	**−1.3**	−1.334	<0.001	0.26	0.16-0.42
**Mechanism**	???/blunt*	**0**	0		1	
	penetrating	**−0.6**	−0.623	<0.001	0.54	0.41-0.70
**Pupil reactivity**	brisk	**+0.2**	0.271	0.001	1.31	1.12-1.53
	???/sluggish*	**0**	0		1	
	fixed	**−1.0**	−1.011	<0.001	0.36	0.30-0.45
**Pupil size**	normal	**+0.2**	0.178	0.014	1.20	1.04-1.38
	???/anisocoria*	**0**	0		1	
	both dilated	**−0.5**	−0.490	<0.001	0.61	0.48-0.78
**Motor function**	normal	**+0.6**	0.609	<0.001	1.84	1.62-2.09
	???/directed*	**0**	0		1	
	non-directed	**−0.4**	−0.378	0.001	0.69	0.55-0.85
	none	**−0.8**	−0.819	<0.001	0.44	0.39-0.50
**CPR**	???/no*	**0**	0		1	
	yes	**−1.8**	−1.752	<0.001	0.17	0.14-0.22
**Blood pressure**	<90	**−0.7**	−0.665	<0.001	0.51	0.44-0.61
	???/90-110*	**0**	0		1	
	111-150	**+0.3**	0.310	<0.001	1.36	1.22-1.53
	>150*	**0**	0			
**INR**	<1.20	**+0.6**	0.637	<0.001	1.89	1.68-2.13
	1.20-1.39	**+0.2**	0.184	0.024	1.20	1.03-1.41
	???/1.40-2.39*	**0**	0		1	
	2.40+	**−0.4**	−0.383	0.001	0.68	0.54-0.86
**Haemoglobin**	12.0+	**+0.4**	0.372	<0.001	1.45	1.30-1.62
	???/7.0-11.9*	**0**	0		1	
	<7.0	**−0.5**	−0.551	<0.001	0.58	0.46-0.73
**Base deficit**	<6.0	**+0.3**	0.267	<0.001	1.31	1.17-1.46
	???/6.0-8.9*	**0**	0		1	
	9.0-14.9	**−0.4**	−0.404	<0.001	0.67	0.53-0.84
	15.0+	**−1.5**	−1.544	<0.001	0.21	0.15-0.30
**Constant**		**+3.6**	3.590	<0.001		

*Reference category. ???, missing value/unknown; ASA, American Society of Anesthesiologists; CI, 95% confidence interval;.CPR, cardiopulmonary resuscitation; INR, international normalized ratio; OR, odds ratio; RISC II, Revised Injury Severity Classification II.

The following variables had been tested for inclusion but finally did not reach sufficient power to be included in the model: type of injury (traffic, high fall, low fall, and so on); change in blood pressure from initial pre-hospital assessment to admission; time from injury to hospital admission, pelvic fracture with relevant blood loss (AIS 5), and Shock Index (SI).

Thirteen variables are needed to calculate the complete RISC II score where the three items derived from the AIS codes were considered as one variable. The average number of missing values was 1.1 in patients documented with the standard data sheet, and 3.1 for patients documented with the reduced data sheet (where pupil size and reactivity were not documented).

**Figure 2:**
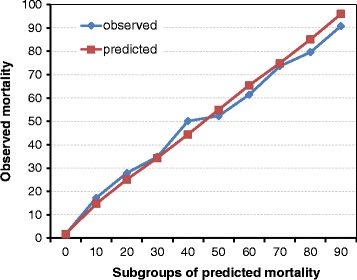
**Observed and predicted mortality rates in 10 subgroups of patients with increasing risk of death based on RISC II.** RISC II, revised injury severity classification II.

**Figure 3:**
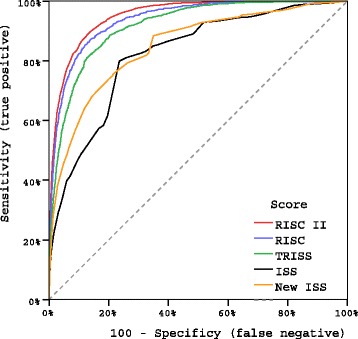
**Receiver operating characteristic curves for RISC II, RISC, TRISS, ISS, and NISS in 17,411 patients from the development dataset with valid data for all five scoring systems.** The areas under the curves are given in Table 4. ISS, Injury Severity Score; NISS, New Injury Severity Score; RISC (II), Revised Injury Severity Classification (II); TRISS, Trauma and Injury Severity Score.

The quality of the RISC II score, as compared to the existing ones, is described in Table 4 in terms of discrimination, precision, and calibration. Comparisons to the original RISC and the TRISS score were limited to those patients with a valid score, respectively (RISC: n = 26,041, 84%; TRISS: 17,411, 56%). The variables used for developing the new score had at least a 20% rate of missing values, created by arbitrary deleting some real observations (except for age and injury severity where completeness was required). If all available information would have been used for calculating the RISC II the results further improved (Table 4). The observed and expected mortality in 10 equal-sized risk bands is given in Figure 2. Figure 3 is a graphical comparison of ROC curves calculated in the subset of patients who had complete information for all considered scores.

**Table 4:** **Quality criteria for the considered scoring systems in the development dataset (TR-DGU 2010 and 2011), and for RISC II in the validation dataset (TR-DGU 2012)**

	**Discrimination**	**Precision**	**Calibration**
	**AUC of ROC curve (95% CI)**	**observed and predicted mortality**	**HL goodness-of-fit statistic**
**All patients, development dataset (n = 30,866)**			
Observed mortality		11.5%	
**RISC II** with 20% missing values per variable	0.943 (0.939-0.946)	11.4%	55.2
**RISC II** with all available data	0.947 (0.944-0.951)	11.6%	55.3
**Patients with RISC, development dataset (n = 26,041)**			
Observed mortality		11.4%	
**RISC II**	0.947 (0.943-0.951)	11.6%	50.8
**RISC**	0.934 (0.929-0.938)	13.5%	233.8
**Patients with TRISS, development dataset (n = 17,411)**			
Observed mortality		10.7%	
**RISC II**	0.953 (0,949-0,957)	11.0%	38.3
**RISC**	0.939 (0.933-0.944)	12.9%	178.6
**TRISS**	0.917 (0.911-0.924)	13.5%	554.4
**NISS**	0.849 (0.839-0.858)		
**ISS**	0.821 (0.811-0.831)		
**All patients, validation dataset (n = 21,918)**			
Observed mortality		10.9%	
**RISC II**	0.951 (0.947-0.954)	11.3%	50.3

The reduced number of patients in the comparative analysis is due to the availability of the RISC and the TRISS score. AUC, area under the curve; ISS, Injury Severity Score; HL, **H**osmer-**L**emeshow; NISS, New Injury Severity Score; RISC (II), Revised Injury Severity Classification (II); ROC, receiver operating characteristic; TR-DGU, TraumaRegister DGU^™^ of the German Trauma Society; TRISS, Trauma and Injury Severity Score.

The patients documented in the TR-DGU in 2012 were used for validation where the same inclusion criteria were used as for the development sample. The patient characteristics of the validation sample are given in Table 1. Discrimination and calibration were even slightly better than in the development dataset, and precision was acceptable (Table 4).

## Discussion

Besides performing benchmarking for hospitals treating severely injured patients trauma registries play an important role in trauma research since classical clinical trials are often difficult to perform, if not even impossible, in the acute care phase [8]. In these situations it is common that the considered patient populations differ considerably. University hospitals treat different patients than small local hospitals; intubated patients were more severely ill than non-intubated patients; transfused patients have a higher risk of death than non-bleeding trauma victims. Furthermore, the injury pattern also are very heterogeneous in terms of location (head, thorax, abdomen, extremities), affected structures (bones, organs, soft tissue), mechanism (blunt, penetrating), and severity. The outcome of trauma victims could therefore only be judged and evaluated if there is some idea about what happens on average to patients with such kind of injuries. Trauma score systems could serve as a helpful tool in these situations.

During the last decades several trauma score systems have been developed, and the knowledge about important prognostic factors and their interaction have considerably increased. Summarizing the anatomical injuries, as done for example by the ISS, was the first attempt to quantify injury severity. The ISS has since become a kind of common language for trauma surgeons and other researchers, and it is the most frequently used trauma score worldwide [9]. This is even more remarkable since it has some serious limitations. Multiple injuries in the same body region are disregarded, and the risk of death from head injuries is known to be underestimated. An ISS of 27 points resulting from three different grade 3 injuries is much less critical than an ISS of 25 from a single grade 5 injury. Furthermore, the ISS depends on the AIS codebook, which repeatedly had been changed and updated.

The NISS was able to address some of the critical points mentioned above by using the three worst injuries irrespective of their location. It had also been included in the first version of the RISC score [4]. However, during the present analyses, it turned out that it makes much more sense to just consider the two worst injuries separately instead of the ISS score. The simple variable 'worst AIS', or 'maximum AIS severity level', received better Nagelkerke's R^2^ values than did any ISS or NISS (continuous or categorical). Similar, Moore *et al*. also found the worst injury to have a better prediction than the ISS in the Trauma Risk Adjustment Model [10]. Furthermore, having the two worst injuries as separate variables in the model not only considerably improves the prediction but also allows to better separate multiple injuries from the isolated ones. The second-worst injury, if only grade 2 or less, improves the outcome in the RISC II model. This has not yet been implemented in any other trauma score.

Some further remarkable aspects were included in the new RISC II score. Children up to the age of 10 years seem to have a better outcome than adults or adolescents. Sex, mechanism of injury, and pre-existing diseases (pre-injury American Society of Anesthesiologists (ASA) classification of physical status), not considered in the original RISC, are now included. GSC is replaced by the simplified motor function of GCS. It has already been described previously by others that this aspect of GCS is the most predictive one (for example the probability of survival models PS12 of the British Trauma Audit and Research Network, (TARN) see [11]). But even more noteworthy is the fact that pupil reactivity and pupil size have now been included. Both aspects are easy to assess and have been recorded since the foundation of the registry. However, only recent analyses showed that their predictive ability was even better than the GCS [12-14]. They independently added prognostic information to the prediction model. This is even more important since about half of the patients did not have these variables recorded (they were not part of the reduced data collection form, see Table 2). The model considerably improved when these variables were added. As a consequence, pupil size and reactivity will soon be added to the reduced basic dataset for all patients.

But the most important design aspect of the RISC II is its handling of missing values. Missing values are a relevant problem in all registries. This is especially true if registry data are collected retrospectively. Source data verification is still a rare exception, if done at all. Patients with missing data in variables needed for score calculation are either excluded from prognostic estimation, or their missing values are imputed based on similar available information, or normal values are simply assumed. This procedure was also used for the original RISC score. The approach chosen here for the new RISC II is different. Missing values will be included in the model as a separate category, specifically as the reference category in logistic regression analysis. This category, by definition, receives a coefficient of zero which does not change the prognosis. If the value is available, then its effect on prognosis might be negative (that is, the prognosis is worsened), or positive in case of normal values, or somewhere in between. However, this procedure could not be applied for every variable since there needs to be a minimum set of reliable information to calculate a basic prognosis. We decided to have age and injury severity (derived from the AIS codes) as the minimum set of information required. If this information was missing no reasonable prognosis seems to be possible. This is, however, no limitation since both variables were obligatory for documentation. For all other variables there is a category for missing variables (indicated as '???' in Table 3).

The big advantage of this approach is that no case has to be excluded from prognostic estimation. The original RISC score excluded patients from calculations if more than half of the information was missing, or if certain missing values could not be replaced. The inclusion of as many cases as possible in risk adjustment analyses could be considered as an important characteristic of a score.

Patients with a maximum injury severity of AIS grade 1 were excluded here. In these patients mortality is very rare, and the very few non-survivor found in this group may have died from other reasons than from trauma (or their documentation of injuries was incomplete). Therefore care should be taken that the RISC II score is not applied to patients with minor injuries.

Other potential outcome predictors were not included here. Respiratory rate (RR), for example, is part of the Revised Trauma Score (RTS), and as such it is also contained in the TRISS score. However, during the development of the original RISC score, RR turned out to have only marginal predictive power. Interestingly, these findings were recently confirmed by Schluter *et al*. who derived actual coefficients for TRISS and RTS on data from the NTDB and from New Zealand [15,16]. He found that the coefficient for RR was by far the smallest one in the revised TRISS model. The most important prognostic information contained in RR seems to be the effect of cardiac arrest (RR = 0), which is covered by the variable CPR in the RISC II.

Hypothermia has repeatedly been demonstrated to be an important predictor of outcome in large samples [17-19]. However, when we tried to add hypothermia to the original RISC model, no additional effect could be demonstrated [20]. This is based on the fact that coagulation is already covered by laboratory values for coagulopathy in the original RISC as well as in the present update. When these coagulation variables were removed hypothermia also became a relevant prognostic factor.

Obesity has been found to be a prognostic factor using TR-DGU data. A body mass index (BMI) of 30 and above increased the risk of death (OR = 1.6). But more interestingly, a BMI below 20 is even more dangerous (OR = 2.1) [21]. Unfortunately, the considerable amount of missing values for weight, and even more for height, in routine patient files led us to remove these items from our dataset in 2009. Thus an additional effect of obesity could not be evaluated here.

We used hospital mortality instead of 30-day mortality. A valid 30-day mortality rate would require to follow-up all patients discharged before that day, which were 84% in our database. It should further be considered that trauma deaths occurring after day 30 mostly affect older people [22]. Using 30-day mortality would thus underestimate this age effect. However, using hospital mortality also has its problems, specifically when a large portion of patients is transferred to other hospitals, or if health care systems encourage such step-down transfers.

There are, of course, some limitations involved in this analysis. As a general weakness data quality in registries is considered inferior to that of clinical trials. To acknowledge this, multiple plausibility and completeness checks have been implemented in the TR-DGU online documentation software. Some measurements may change quickly (for example, blood pressure (BP)), others like base deficit were routinely measured but frequently not documented, and again others could have been influenced by pre-hospital treatment (for example, volume administration, catecholamines). During the analysis we preferred categorical variables rather than continuous ones (or derived functions thereof), knowing that ‘exact’ measurements are not as exact as they seem to be. Using categories instead is much more robust, at the cost of disregarding details. We also avoided including interaction terms, both because of their limited influence in prediction models as well as the desired simplicity of the final model.

**Figure 4:**
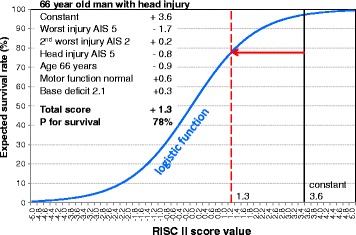
**Example for the application of the new RISC II score.** The variables not listed here got 0 points and thus did not change the prognosis.

It was the initial aim to develop an updated score, which is easier to use than the original RISC, with better discrimination and precision, and without excluding cases from prognostic estimation. All these goals have been reached. Figure 4 demonstrates a sample application of the new score. Further external evaluations in other datasets outside the TR-DGU will have to show the usefulness of this tool. The original RISC score based on data from the 1990s was able to show that the observed mortality fell about 2% below the prognosis, based on advances in medical and surgical treatment. Hopefully, the new RISC II will observe a similar progress in future.

## Conclusions

Adjustment of outcome is mandatory in case of heterogeneous populations like severe trauma patients. Furthermore, with an increasing knowledge about prognostic factors and improvements of therapeutic strategies, updated scores are required. The update of the Revised Injury Severity Classification score (RISC II) includes several new predictors, like pupil size and reactivity, but also an innovative type of management of missing values. First validation studies show that it is superior to the existing scoring systems, including the original RISC.

## Key message

- New prognostic factors have been included into the updated RISC II: pupil size and reactivity, pre-trauma ASA, gender, and laboratory values on admission
- Injury severity is best presented with the worst and the second worst injury only, plus additional points for head injury
- Missing values are no longer excluded or imputed but included in the model
- The quality of a predictive scoring system is measured by discrimination, precision, and calibration; the new RISC II was able to improve all of these, compared with RISC and TRISS.

## Abbreviations

???: missing value
AIS: Abbreviated Injury Scale
ASA: American Society of Anesthesiologists (classification of physical status)
AUC: area under the ROC curve
BMI: body mass index
BP: blood pressure
CI: 95% confidence interval
CPR: cardiopulmonary resuscitation
DGU: German Trauma Society (Deutsche Gesellschaft für Unfallchirurgie)
ECS: Eppendorf-Cologne scale
GCS: Glasgow Coma Scale
HL: Hosmer-Lemeshow goodness-of-fit statistic
INR: international normalized ratio
ISS: Injury Severity Score
NISS: New Injury Severity Score
NTDB: National Trauma Data Bank (United States)
OR: odds ratio
PS12: actual prediction of survival model of TARN
RISC: Revised Injury Severity Classification
ROC: receiver operating characteristic
RR: respiratory rate
RTS: Revised Trauma Score
SI: Shock Index
SD: standard deviation
TARN: Trauma Audit and Research Network (United Kingdom trauma registry)
TR-DGU: TraumaRegister DGU™ of the German Trauma Society
TRISS: Trauma and Injury Severity Score

## References

[1] Trunkey DD, Siegel J, Baker SP, Gennarelli TA (1983). Panel: Current status of trauma severity indices. J Trauma.

[2] Baker SP, O'Neill B, Haddon W, Long WB (1974). The Injury Severity Score: a method for describing patients with multiple injuries and evaluating emergency care. J Trauma.

[3] Champion HR, Copes WS, Sacco WJ, Lawnick MM, Keast SL, Bain LW, Flanagan ME, Frey CF (1990). The Major Trauma Outcome Study: establishing national norms for trauma care. J Trauma.

[4] Lefering R (2009). Development and validation of the Revised Injury Severity Classification score for severely injured patients. Europ J Trauma Emerg Med.

[5] Ringdal KJ, Coates TJ, Lefering R, di Bartolomeo S, Steen PO, Røise O, Handolin L, Lossius HM, panel UTe: **The Utstein template for uniform reporting of data following major trauma: a joint revision by SCANTEM, TARN, DGU-TR and RITG.** *Scand J Trauma Resuscitation Emerg Med* 2008, **16:**7. 10.1186/1757-7241-16-7 PMC2568949 18957069

[6] **TraumaRegister DGU® of the German Trauma Society.** [www.traumaregister.de].

[7] Osler T, Baker SP, Long W (1997). A modification of the Injury Severity Score that both improves accuracy and simplifies scoring. J Trauma.

[8] Lefering R, Ruchholtz S (2012). Trauma registries in Europe (Editorial). Europ J Trauma Emerg Med.

[9] Lefering R (2002). Trauma score systems for quality assessment. Europ J Trauma Emerg Med.

[10] Moore L, Lavoie A, Turgeon AF, Abdous B, Le Sage N, Emond M, Libermann M, Bergeron E (2009). The trauma risk adjustment model. A new model for evaluating trauma care. Ann Surg.

[11] Bouamra O, Wrotchford A, Hollis S, Vail A, Woodford M, Lecky F (2006). Outcome prediction in trauma. Injury.

[12] Hoffmann M, Lehmann W, Rueger JM, Lefering R, Trauma Registry of DGU (2012). Introduction of a novel trauma scale. J Trauma.

[13] Hoffmann M, Lefering R, Rueger JM, Kolb JP, Izbicki JR, Ruecker AH, Rupprecht M, Lehmann W, Trauma Registry of DGU (2012). Pupil evaluation in addition to the Glasgow Coma Scale (GCS) components in traumatic brain injury. Br J Surg.

[14] Huber-Wagner S, Stegmaier J, Mathonia P, Paffrath T, Euler E, Mutschler M, Kanz KG, Lefering R (2010). The sequential trauma score - a new instrument for the sequential mortality prediction in major trauma. Eur J Med Res.

[15] Schluter PJ (2011). Trauma and Injury Severity Score (TRISS): is it time for variable re-categorisation and re-characterisation?. Injury.

[16] Schluter JP (2011). The Trauma and Injury Severity Score (TRISS) revised. Injury.

[17] Martin RS, Kilgo PD, Miller PR, Hoth JJ, Meredith JW, Chang MC (2005). Injury-associated hypothermia: an analysis of the 2004 National Trauma Data Bank. Shock.

[18] Shafi S, Elliott AC, Gentilello L (2005). Is hypothermia simply a marker of shock and injury severity or an independent risk factor for mortality in trauma patients? Analysis of a large national trauma registry. J Trauma.

[19] Wang HE, Callaway CW, Peitzman AB, Tisherman SA (2005). Admission hypothermia and outcome after major trauma. Crit Care Med.

[20] Trentzsch H, Huber-Wagner S, Hildebrand F, Kanz KG, Faist E, Piltz S, Lefering R, Trauma Registry of DGU (2012). Hypothermia for prediction of death in severely injured blunt trauma patients. Shock.

[21] Hoffmann M, Lefering R, Gruber-Rathmann M, Rueger JM, Lehmann W, Trauma Registry of DGU (2012). The impact of BMI on polytrauma outcome. Injury.

[22] Lefering R, Paffrath T, Bouamra O, Coats TJ, Woodford M, Jenks T, Wafaisade A, Nienaber U, Lecky F (2012). Epidemiology of in-hospital trauma deaths. Europ J Trauma Emerg Med.

